# Association of Nighttime Sleep Duration at 1.5 Years With Height at 3 Years: The Japan Environment and Children's Study

**DOI:** 10.1210/clinem/dgae647

**Published:** 2024-09-17

**Authors:** Masanobu Kawai, Sachiko Baba, Kanami Tanigawa, Satoyo Ikehara, Ryo Kawasaki, Hiroyasu Iso

**Affiliations:** Department of Molecular Genetics and Endocrinology, Research Institute, Osaka Women's and Children's Hospital, Izumi, Osaka 594-1101, Japan; Department of Gastroenterology, Nutrition, and Endocrinology, Osaka Women's and Children's Hospital, Izumi, Osaka 594-1101, Japan; Maternal and Child Health Information Center, Osaka Women's and Children's Hospital, Izumi, Osaka 594-1101, Japan; Maternal and Child Health Information Center, Osaka Women's and Children's Hospital, Izumi, Osaka 594-1101, Japan; Department of Social Medicine, Osaka University Graduate School of Medicine, Suita, Osaka 565-0871, Japan; Department of Social Medicine, Osaka University Graduate School of Medicine, Suita, Osaka 565-0871, Japan; Institute for Global Health Policy Research, Bureau of International Health Cooperation, National Center for Global Health and Medicine, Shinjuku-ku, Tokyo 162-8655, Japan

**Keywords:** nighttime sleep duration, body height, birth cohort, child

## Abstract

**Context:**

Adequate nighttime sleep duration has been considered beneficial for linear growth in children; however, there is limited and conflicting evidence regarding the association between sleep duration and subsequent linear growth.

**Objective:**

To investigate the association between sleep duration at 1.5 years and height at 3 years of age.

**Methods:**

The Japan Environment and Children's Study is a nationwide prospective birth cohort study. Data from 52 140 term singleton births born at an appropriate-for-gestational age without background disorders that could potentially affect linear growth in the analyses were included. Nighttime and total sleep durations were calculated based on a self-administered questionnaire completed by caregivers. Tall stature was defined as height at or above the 75th percentile among participants.

**Results:**

After adjustment for height at 1.5 years, sex, monthly age, mother's height, presence of siblings at 1.5 years, environmental tobacco smoke at 1.5 years, daily TV/DVD screen time at 2 years, attendance at nursery at 2 years, household annual income at birth, and parents’ educational status, multivariate odds ratio (95% CI) for tall stature at 3 years were 1.09 (1.01-1.17), 1.09 (1.01-1.17), and 1.25 (1.14-1.37) for 9.5 or 10, 10.5 or 11, and ≥ 11.5 hours of nighttime sleep duration at 1.5 years, respectively, compared with those with ≤ 9 hours sleep (*P* for trend <.0001). Total sleep duration was not associated with tall stature.

**Conclusion:**

This study underscores the importance of nighttime sleep duration, not total sleep duration, in the linear growth of very young children.

Linear growth during childhood is influenced by many factors, including nutritional, genetic, hormonal, environmental, and social elements, and dysregulation of these processes has been associated with impaired linear growth (1-4). Many genes that contribute to short stature have been identified (3). Hormonal regulation plays a key role in linear growth, as evidenced by impaired growth in individuals with growth hormone (GH) deficiency (1, 2). Despite substantial evidence supporting the genetic, endocrinological, and nutritional regulation of linear growth, the mechanisms through which environmental and social factors, such as sleep duration, influence linear growth remain poorly understood.

Sleep is crucial for human health, and its disruption, such as a short sleep duration, has been linked to the development of various diseases (5, 6). Short sleep duration has been associated with metabolic complications, including childhood obesity (6, 7). Given the importance of adequate sleep for human health and the established link between slow-wave sleep and GH secretion (8-10), sleep duration is potentially associated with linear growth. However, there is limited evidence on this topic, and the conclusions drawn in previous studies have been conflicting (11-17). These inconsistencies are, in part, due to the relatively small sample sizes of previous studies, as well as variations in the ages at outcome evaluation and consideration of appropriate confounding variables.

To address these limitations, we used a large-scale birth cohort study consisting of 104 062 fetal records to investigate the association between sleep duration and subsequent height outcomes during early childhood.

## Methods

### The Japan Environment and Children's Study and Ethical Considerations

The Japan Environment and Children's Study (JECS) is a nationwide birth cohort study designed to investigate the impact of environmental and lifestyle factors as well as medical and psychosocial conditions during pregnancy and childhood on the health and development of offspring. The JECS was established in 2011 as a collaboration between the Program Office (National Institute for Environmental Studies), Medical Support Center (National Center for Child Health and Development), and 15 Regional Centers. Pregnant women were recruited between January 2011 and March 2014 from cooperating healthcare providers or municipal offices. A total of 104 062 fetuses were registered during the recruitment period.

The JECS protocol was reviewed and approved by the Ministry of the Environment's Institutional Review Board on Epidemiological Studies (No. 100 910 001) and the Ethics Committees of all participating institutions. Written informed consent was obtained from all participants. Descriptions of the JECS have been reported previously (18, 19).

### Participant Enrollment

**Figure 1.**
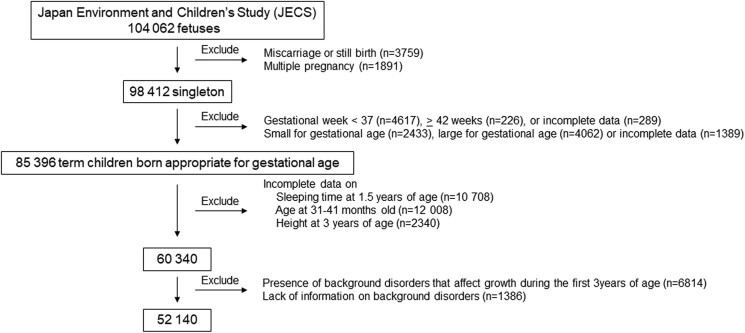
Flowchart of study enrollment.

A self-administered questionnaire-based survey was completed during pregnancy, 1 month after birth, and every 6 months until 3 years of age. In addition, maternal anthropometric data before pregnancy and perinatal outcomes, including sex, multiple pregnancies, gestational age (in weeks) at birth, and birth weight and length, were collected through data transcription from medical records. Data transcription was performed by physicians, midwives, nurses, and/or Research Coordinators. The *jecs-ta-20190930-qsn dataset*, released in October 2019, was used in this study. Participant enrollment is illustrated in Fig. 1. Among the 104 062 fetuses, miscarriages or still births (n = 3759) and multiple pregnancies (n = 1891) were excluded. As such, 98 412 singleton live births were included and, among these, preterm birth (< 37 weeks’ gestation [n = 4617]), post-term birth (≥ 42 weeks’ gestation [n = 226]), or incomplete data (n = 289), those born small for gestational age (n = 2433), large for gestational age (n = 4062), or incomplete data (n = 1389) were excluded. Small or large for gestational age was defined as either of birth weight standard deviation score (SDS) or length SDS < −2.0 SD or > 2.0 SD, respectively, based on the sex-specific standard data on birth weight and length stratified according to gestational age in the Japanese population (20). Among the remaining 85 396 term children born appropriate for gestational age, those with incomplete data regarding sleep duration at 1.5 years of age (n = 10 708) and 31 to 41 months of age (n = 12 008), and height (n = 2340) at 3 years of age were excluded. Information regarding sex was available for all participants at this stage. Incomplete data regarding age included outliers in which age was < 31 months or > 41 months. Incomplete data regarding height also included outliers, which was defined as those with height SDS being outside of the range of the mean ± 5 SD at 3 years of age. Among the 60 340 participants, those with background disorders that potentially affected growth during the first 3 years of life (n = 6814) or those with missing data regarding background disorders (n = 1386) were excluded. Excluded background disorders included congenital anomalies, endocrine disorders (diabetes mellitus, thyroid disorders, GH deficiency, precocious puberty), neurological disorders (epilepsy, cerebral palsy), hematological disorders (leukemia, anemia), tumors (cranial tumors, neuroblastoma, other malignant tumors), immunodeficiency, encephalopathy, meningitis, cardiac diseases (myocarditis, infective endocarditis, arrythmia), hepatitis, motor and intellectual disabilities, autism spectrum disorder, and eating disorders. Ultimately, data from 52 140 participants were included in the study (Fig. 1).

### Exposure

#### Sleep time

Sleep duration was calculated based on a questionnaire completed at 1.5 years of age. Parents were asked to mark boxes representing the intervals of 0.5 hours from 12:00 Am to 12:00 Am the next day, when their offspring were asleep; therefore, sleep duration was documented at 0.5 hours intervals. Wake-up and bedtime were defined according to the criteria outlined in the nationwide survey entitled “Survey on time use and leisure activities” among the general population aged 10 years or older, which was conducted by the Statistics Bureau, Ministry of Internal Affairs and Communications of Japan (https://www.stat.go.jp/english/data/shakai/2021/pdf/kaisetsua-e.pdf). Briefly, wake-up time was defined as the time when uninterrupted sleep > 1 hour ended between 12:00 Am and 12:00 Pm, whereas bedtime was defined as the time when continuous sleep > 1 hour commenced after 5:00 Pm. In cases with multiple continuous sleep periods > 1 hour, the starting time of the longer sleep period was used as nighttime sleep. When sleep durations were the same among multiple sleep periods, the starting time of the earlier sleep period was used. When the duration between continuous sleep periods > 1 hour was 0.5 hours, these periods were not considered separate and were treated as one continuous sleep. Nighttime sleep duration was defined as the duration between bedtime and wake-up time, categorized as follows: ≤ 9 hours, 9.5 or 10 hours, 10.5 or 11 hour, and ≥ 11.5 hours. Total sleep time represented the sum of sleep times within a 24-hour period, starting from 12:00 Am to 12:00 Am the next day, and was categorized as follows: ≤ 11 hours, 11.5 hours, 12 hours, 12.5 hours, 13 hours, and ≥ 13.5 hours.

### Outcome

#### Height at 3 years of age

Anthropometric parameters, including height, primarily measured at nursery schools, municipal health centers, and medical facilities, and transcribed by caregivers, were collected based on a questionnaire obtained at 3 years of age. Sex- and age-adjusted height SDS (HT-SDS) were calculated based on normal growth standards for Japanese children from a national survey conducted in 2000 (21). Tall stature was defined as HT-SDS ≥ 0.50, which was equivalent to the ≥ 75th percentile among participants, whereas short stature was defined as HT-SDS below −1.08, corresponding to < 25th percentile among participants. Participants with HT-SDS, who did not meet either criterion, were categorized as normal stature.

### Statistical Analysis

Analyses were conducted separately for nighttime and total sleep duration. Categorical variables are expressed as category proportions, and continuous variables, such as HT-SDS at 3 years and monthly age, are expressed as mean (SD). Multivariable logistic regression analyses were performed to calculate odds ratio (OR) and corresponding 95% CI for the association between sleep time at 1.5 years and height at 3 years of age, using the lowest sleep time group as the reference. Adjusted OR was calculated considering the variables mentioned below, whereas crude OR was calculated without using these variables. Model 1 was adjusted for variables known to affect height at 3 years, including height at 1.5 years (tall, normal, or short stature), sex (male or female), and monthly age at 3 years. Mother's height (< 150.0, 150.0-159.9, 160.0-169.9 or ≥170.0 cm) was also included as a variable due to its strong genetic influence on offspring height. Father's height was not included because it was not available for 46.7% of participants. Model 2 further included the presence of sibling(s) at 1.5 years (yes or no), environmental tobacco smoke at 1.5 years (yes or no), daily television (TV) and/or digital video disc (DVD) screen time at 2 years (0, 0.01-0.99, 1.00-1.99, 2.00-3.99, or ≥ 4.00 hours), attendance at nursery at 2 years of age (yes or no), annual household income during pregnancy (< 2000, 2000-3999, 4000-5999, 6000-7999 or ≥ 8000 thousand yen), and mother's and father's educational status (junior high school, high school, technical college/vocational school/junior college or university/graduate school). Missing data for variables (0.0% to 9.4% for each) were included as categorical variables in the model. A trend test was performed to determine the average sleep duration in each group. Additionally, stratified analyses according to dichotomous variables (sex, the presence of sibling[s] at 1.5 years, environmental tobacco smoke at 1.5 years, or attendance to nursery at 2 years on height at 3 years) were performed to examine effect modification in the associations between nighttime sleep duration at 1.5 years and height at 3 years. The cross-product terms of average sleep duration and these variables were added to the multivariate logistic regression models and tested for their interactions. Statistical analyses were performed using EZR (Saitama Medical Center, Jichi Medical University, Saitama, Japan) with a graphical user interface for R (R Foundation for Statistical Computing, Vienna, Austria). Differences with *P* < .05 were considered to be statistically significant.

## Results

### Subject Characteristics

The number of subjects and their characteristics according to nighttime and total sleep durations among 52 140 eligible children are summarized in Table 1. Individuals with nighttime sleep duration of 9.5 hours or 10 hours were the most prevalent, whereas those with ≥ 11.5 hours were the lowest. Tall stature was observed in 28.0% of subjects with nighttime sleep duration ≥ 11.5 hours, whereas it was 23.8% in the group with least nighttime sleep duration. In contrast, tall stature was noted in 25.8% of subjects with total sleep duration of 13.5 hours, whereas it was 24.3% in the group with the lowest total sleep duration.

**Table 1.** Characteristics of participants according to nighttime sleep duration at 1.5 years (n = 52 140)

		Total number (n = 52 140)	Nighttime sleep duration (hours)				Total sleep duration (hours)			
			9 or less	9.5 or 10	10.5 or 11	11.5 or more	11 or less	11.5 or 12	12.5 or 13	13.5 or more
			n = 6809	n = 21 474	n = 18 447	n = 5 410	n = 9617	n = 11 023	n = 18 076	n = 13 424
HT-SDS at 3 years, mean (SD)		−0.14 (0.98)	−0.20 (0.98)	−0.16 (0.97)	−0.12 (0.99)	−0.10 (1.03)	−0.16 (0.98)	−0.15 (0.97)	−0.14 (0.99)	−0.13 (0.99)
Height at 3 years	Tall*^a^*	13 002 (24.9%)	1552 (23.8%)	5205 (24.2%)	4730 (25.6%)	1515 (28.0%)	2341 (24.3%)	2693 (24.4%)	4508 (24.9%)	3460 (25.8%)
	Short*^a^*	12 404 (23.8%)	1704 (25.0%)	5192 (24.2%)	4242 (23.0%)	1266 (23.4%)	2310 (24.0%)	2627 (23.8%)	4303(23.8%)	3164 (23.6%)
	Normal	26 734 (51.3%)	3553 (52.2%)	11 077 (51.6%)	9475 (51.4%)	2629 (48.6%)	4966 (51.6%)	5703 (51.7%)	9265 (51.3%)	6800 (50.7%)
Height at 1.5 years	Tall*^a^*	12 008 (23.0%)	1500 (22.0%)	4759 (22.2%)	4437 (24.1%)	1312 (24.3%)	2127 (22.1%)	2523 (22.9%)	4152 (23.0%)	3206 (23.9%)
	Short*^a^*	11 023 (21.1%)	1547 (22.7%)	4637 (21.6%)	3754 (20.4%)	1085 (20.1%)	2120 (22.0%)	2351 (21.3%)	3821 (21.1%)	2731 (20.3%)
	Normal	24 204 (46.4%)	3179 (46.7%)	10 244 (47.7%)	8381 (45.4%)	2400 (44.4%)	4393 (45.7%)	5196 (47.1%)	8457 (46.8%)	6158 (45.9%)
	Unknown	4905 (9.4%)	583 (8.6%)	1834 (8.5%)	1875 (10.2%)	613 (11.3%)	977 (10.2%)	953 (8.6%)	1646 (9.1%)	1329 (9.9%)
Sex	Male	26 006 (49.9%)	3535 (51.9%)	10 835 (50.5%)	9041 (49.0%)	2595 (48.0%)	4737 (49.3%)	5427(49.2%)	8968 (49.6%)	6874 (51.2%)
	Female	26 134 (50.1%)	3274 (48.1%)	10 639 (49.5%)	9406 (51.0%)	2815 (52.0%)	4880 (50.7%)	5596 (50.8%)	9108 (50.4%)	6550 (48.8%)
Age at 3 years (months), mean (SD)		35.24 (1.11)	35.24 (1.09)	35.23 (1.09)	35.25 (1.11)	35.26 (1.18)	35.23 (1.11)	35.25 (1.12)	35.23 (1.08)	35.25 (1.13)
Mother's height, cm	<150.0	2179 (4.2%)	281 (4.1%)	899 (4.2%)	751 (4.1%)	248 (4.6%)	398 (4.1%)	480 (4.4%)	770 (4.3%)	531 (4.0%)
	150.0-159.9	28 885 (55.4%)	3837 (56.4%)	11 934 (55.6%)	10 136 (54.9%)	2978 (55.0%)	5379 (55.9%)	6136 (55.7%)	9980 (55.2%)	7390 (55.1%)
	160.0-169.9	20 090 (38.5%)	2573 (37.8%)	8246 (38.4%)	7190 (39.0%)	2081 (38.5%)	3670 (38.2%)	4221 (38.3%)	6977 (38.6%)	5222 (38.9%)
	≥170.0	980 (1.9%)	116 (1.7%)	393 (1.8%)	368 (2.0%)	103 (1.9%)	168 (1.7%)	185 (1.7%)	348 (1.9%)	279 (2.1%)
	Unknown	6 (0.0%)	2 (0.0%)	2 (0.0%)	2 (0.0%)	0 (0.0%)	2 (0.0%)	1 (0.0%)	1 (0.0%)	2 (0.0%)
Presence of sibling(s) at 1.5 years	Yes	29 010 (55.6%)	3895 (57.2%)	12 382 (57.7%)	9980 (54.1%)	2753 (50.9%)	5236 (54.4%)	6248 (56.7%)	10 109 (55.9%)	7417 (55.3%)
	No	23 130 (44.4%)	2914 (42.8%)	9092 (42.3%)	8467 (45.9%)	2657 (49.1%)	4381 (45.6%)	4775 (43.3%)	7967 (44.1%)	6007 (44.7%)
	Unknown	0 (0.0%)	0 (0.0%)	0 (0.0%)	0 (0.0%)	0 (0.0%)	0 (0.0%)	0 (0.0%)	0 (0.0%)	0 (0.0%)
Environmental tobacco smoke at 1.5 years	Yes	15 783 (30.3%)	2088 (30.7%)	6484 (30.2%)	5496 (29.8%)	1715 (31.7%)	6532 (67.9%)	7623 (69.2%)	12 397 (68.6%)	8866 (66.0%)
	No	35 418 (67.9%)	4583 (67.3%)	14 614 (68.1%)	12 615 (68.4%)	3606 (66.7%)	2896 (30.1%)	3189 (28.9%)	5359 (29.6%)	4339 (32.3%)
	Unknown	939 (1.8%)	138 (2.0%)	376 (1.8%)	336 (1.8%)	89 (1.6%)	189 (2.0%)	211 (1.9%)	320 (1.8%)	219 (1.6%)
Daily TV and/or DVD screen time at 2 years, hours	0	1009 (1.9%)	123 (1.8%)	412 (1.9%)	386 (2.1%)	88 (1.6%)	222 (2.3%)	201 (1.8%)	325 (1.8%)	261 (1.9%)
	0.01-0.99	14 399 (27.6%)	1805 (26.5%)	6187 (28.8%)	5058 (27.4%)	1349 (24.9%)	2813 (29.3%)	2897 (26.3%)	4954 (27.4%)	3735 (27.8%)
	1.00-1.99	22 288 (42.7%)	2795 (41.0%)	9232 (43.0%)	7956 (43.1%)	2305 (42.6%)	4034 (41.9%)	4730 (42.9%)	7792 (43.1%)	5732 (42.7%)
	2.00-3.99	12 082 (23.2%)	1700 (25.0%)	4799 (22.3%)	4240 (23.0%)	1343 (24.8%)	2105 (21.9%)	2662 (24.1%)	4246 (23.5%)	3069 (22.9%)
	≥4.00	2304 (4.4%)	379 (5.6%)	812 (3.8%)	794 (4.3%)	319 (5.9%)	433 (4.5%)	517 (4.7%)	739 (4.1%)	615 (4.6%)
	Unknown	58 (0.1%)	7 (0.1%)	32 (0.1%)	13 (0.1%)	6 (0.1%)	10 (0.1%)	16 (0.1%)	20 (0.1%)	12 (0.1%)
Attendance to nursery at 2 years	Yes	26 468 (50.8%)	3978 (58.4%)	12 699 (59.1%)	8111 (44.0%)	1680 (31.1%)	5671 (59.0%)	5561 (50.4%)	9093 (50.3%)	6143 (45.8%)
	No	25 284 (48.5%)	2787 (40.9%)	8588 (40.0%)	10 211 (55.4%)	3698 (68.4%)	3849 (40.0%)	5380 (48.8%)	8851 (49.0%)	7204 (53.7%)
	Unknown	388 (0.7%)	44 (0.6%)	187 (0.9%)	125 (0.7%)	32 (0.6%)	97 (1.0%)	82 (0.7%)	132 (0.7%)	77 (0.6%)
Household annual income, thousand yen	<2000	2191 (4.2%)	276 (4.1%)	859 (4.0%)	783 (4.2%)	273 (5.0%)	419 (4.4%)	418 (3.8%)	744 (4.1%)	610 (4.5%)
	2000-3999	16 068 (30.8%)	2053 (30.2%)	6334 (29.5%)	5844 (31.7%)	1837 (33.4%)	2937 (30.5%)	3346 (30.4%)	5604 (31.0%)	4181 (31.1%)
	4000-5999	16 659 (32.0%)	2040 (30.0%)	6856 (31.9%)	6016 (32.6%)	1747 (32.3%)	2998 (31.2%)	3578 (32.5%)	5786 (32.0%)	4297 (32.0%)
	6000-7999	8335 (16.0%)	1202 (17.7%)	3598 (16.8%)	2831 (13.0%)	704 (13.0%)	1587 (16.5%)	1792 (16.3%)	2882 (15.9%)	2074 (15.4%)
	≥8000	5688 (10.9%)	850 (12.5%)	2524 (11.8%)	1820 (9.1%)	494 (9.1%)	1095 (11.4%)	1244 (11.3%)	1995 (11.0%)	1354 (10.1%)
	Unknown	3199 (6.1%)	388 (5.7%)	1303 (6.1%)	1153 (6.6%)	355 (4.3%)	581 (6.0%)	645 (5.9%)	1065 (5.9%)	908 (6.8%)
Mother's educational status	Junior high school	1597 (3.1%)	192 (2.8%)	602 (2.8%)	570 (3.1%)	233 (4.3%)	322 (3.3%)	326 (3.0%)	508 (2.8%)	441 (3.3%)
	High school	14 992 (28.8%)	1974 (29.0%)	6113 (28.5%)	5247 (28.4%)	1658 (30.6%)	2710 (28.2%)	3138 (28.5%)	5061 (28.0%)	4083 (30.4%)
	Higher professional school/Junior college	22 534 (43.2%)	2917 (42.8%)	9447 (44.0%)	7971 (43.2%)	2199 (40.6%)	4146 (43.1%)	4611 (41.8%)	7872 (43.5%)	5905 (44.0%)
	College/University/Graduate school	12 667 (24.3%)	1682 (24.7%)	5185 (24.1%)	4522 (24.5%)	1278 (23.6%)	2362 (24.6%)	2889 (26.2%)	4508 (24.9%)	2908 (21.7%)
	Unknown	350 (0.7%)	44 (0.6%)	127 (0.6%)	137 (0.7%)	42 (0.8%)	77 (0.8%)	59 (0.5%)	127 (0.7%)	87 (0.6%)
Father's educational status	Junior high school	2959 (5.7%)	402 (5.9%)	1185 (5.5%)	1016 (5.5%)	356 (6.6%)	623 (6.5%)	584 (5.3%)	948 (5.2%)	804 (6.0%)
	High school	17 953 (34.4%)	2454 (36.0%)	7541 (35.1%)	6181 (33.5%)	1777 (32.8%)	3333 (34.7%)	3738 (33.9%)	6128 (33.9%)	4754 (35.4%)
	Higher professional school/Junior college	11 850 (22.7%)	1547 (22.7%)	4937 (23.0%)	4193 (22.7%)	1173 (21.7%)	2216 (23.0%)	2465 (22.4%)	4122 (22.8%)	3047 (22.7%)
	College/University/Graduate school	18 775 (36.0%)	2334 (34.3%)	7579 (35.3%)	6840 (37.1%)	2022 (37.4%)	3322 (34.5%)	4124 (37.4%)	6676 (36.9%)	4653 (34.7%)
	Unknown	603 (1.2%)	72 (1.1%)	232 (1.1%)	217 (1.2%)	82 (1.5%)	123 (1.3%)	112 (1.0%)	202 (1.1%)	166 (1.2%)

Due to the rounding nature of calculations, the total sum of each percentage does not always add up to 100%.

^*a*^Tall stature was defined as height at or above 75th percentile among participants, whereas short stature was defined as height below the 25th percentile.

### Nighttime Sleep Duration and Subsequent Tall Stature

Longer nighttime sleep duration was positively associated with tall stature (crude OR 1.32 [95% CI, 1.21-1.43]) in subjects with sleep time ≥ 11.5 hours compared to those with ≤ 9 hours (Table 2). After full adjustment (Model 2), multivariable ORs (95% CIs) for tall stature were 1.09 (1.01-1.17), 1.09 (1.01-1.17), and 1.25 (1.14-1.37), in those with 9.5 or 10 hours, 10.5 or 11 hour, and ≥ 11.5 hours of nighttime sleep duration, respectively, and we observed a linear trend between nighttime sleep duration and tall stature (*P* < .0001). Longer total sleep duration, which included daytime naps, was positively associated with tall stature in the crude analysis (*P* for trend = .008), but not in the multivariate analyses (Model 1 or Model 2). Longer nighttime or total sleep duration was not associated with short stature in the multivariate analyses.

**Table 2.** Odds ratios (95% CI) of tall or short stature at 3 years according to sleep duration at 1.5 years (n = 52 140)

		Nighttime sleep duration (hours)					Total sleep duration (hours)				
		9 or less	9.5 or 10	10.5 or 11	11.5 or more	*P* for trend	11 or less	11.5 or 12	12.5 or 13	13.5 or more	*P* for trend
	Number at risk	6809	21 474	18 447	5410		9617	11 023	18 706	13 424	
Tall stature*^a^*	Number of cases	1552	5205	4730	1515		2341	2693	4508	3460	
	OR (95% CI) (Crude)	Reference	1.08 (1.02-1.16)	1.17 (1.09-1.25)	1.32 (1.21-1.43)	<.0001	Reference	1.00 (0.94-1.07)	1.03 (0.98-1.09)	1.08 (1.02-1.15)	.008
	OR (95% CI) (Model 1)	Reference	1.09 (1.01-1.17)	1.10 (1.02-1.19)	1.29 (1.17-1.41)	<.0001	Reference	0.98 (0.92-1.06)	1.01 (0.94-1.07)	1.03 (0.96-1.10)	.33
	OR (95% CI) (Model 2)	Reference	1.09 (1.01-1.17)	1.09 (1.01-1.17)	1.25 (1.14-1.37)	<.0001	Reference	0.98 (0.91-1.05)	1.00 (0.94-1.07)	1.02 (0.95-1.09)	.51
Short stature*^a^*	Number of cases	1704	5192	4242	1266		2310	2627	4303	3164	
	OR (95% CI) (Crude)	Reference	0.96 (0.90-1.02)	0.90 (0.84-0.95)	0.92 (0.84-1.00)	.001	Reference	0.99 (0.93- 1.06)	0.99 (0.93-1.05)	0.98 (0.92-1.04)	.44
	OR (95% CI) (Model 1)	Reference	0.98 (0.91-1.05)	0.95 (0.88-1.02)	0.98 (0.89-1.08)	0.30	Reference	1.02 (0.95-1.10)	1.03 (0.96-1.10)	1.04 (0.97-1.12)	.26
	OR (95% CI) (Model 2)	Reference	0.98 (0.91-1.05)	0.95 (0.88-1.02)	0.99 (0.90-1.09)	.39	Reference	1.02 (0.95-1.10)	1.03 (0.96-1.10)	1.04 (0.97-1.12)	.26

Model 1: Adjusted for height at 1.5 years, sex, monthly age at 3 years, and mother's height.

Model 2: Model 1 + the presence of sibling(s) at 1.5 years, environmental tobacco smoke at 1.5 years, daily TV and/or DVD screen time at 2 years, attendance to nursery at 2 years, household annual income at birth, and mother's and father's educational status.

^*a*^Tall stature was defined as height at or above 75th percentile among participants, whereas short stature was defined as height below the 25th percentile. The percentage of individuals who meet this definition among all participants was evaluated.

### Stratified Analysis According to Sex, Presence of Siblings, Environmental Tobacco Smoke, and Attendance at Nursery

**Figure 2.**
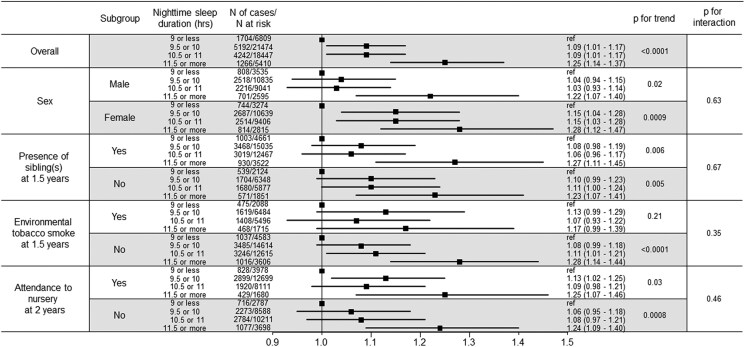
Forest plot showing the odds ratios (95% CI), *P* value for trend, and *P* value for interaction of nighttime sleep duration and tall stature in the different groups.

Additional analyses stratified according to sex, presence of siblings at 1.5 years, environmental tobacco smoke at 1.5 years, and attendance at the nursery at 2 years were performed (Fig. 2). Generally, longer nighttime sleep durations were associated with tall stature in each stratum. No interaction was found between these variables in the association between nighttime sleep duration and tall stature.

## Discussion

In this nationwide birth cohort study, we found a positive association between nighttime sleep duration and subsequent tall stature in young children. Notably, we also revealed a lack of association between total sleep duration and subsequent tall stature, which underscores the importance of nighttime sleep on linear growth. We also performed stratified analyses according to sex, presence of sibling(s), environmental tobacco smoke, and attendance at nursery. However, no differences were observed in the above associations based on these factors.

Previous studies have been conducted with relatively small numbers of subjects of varying ages. A cross-sectional study in China reported that short sleep duration was associated with shorter height in 143 lean children (13). A birth cohort study involving 899 children in Singapore revealed a positive association between sleep duration at 3 months and height at 2 years of age (14). In contrast, a cross-sectional study involving 5145 children 5 to 11 years of age in England and Scotland demonstrated weak and inverse associations between sleep duration and growth (11). A cohort study involving 305 Swiss children reported no association between sleep duration and height at any age between 1 and 10 years (12). Thus, findings from previous investigations have been inconsistent. In this context, the current study provides additional evidence supporting the association between sleep duration and linear growth in very young children.

The mechanisms by which longer sleep duration at night, but not total sleep duration, is associated with better linear growth may be multifactorial; however, GH secretion during night-time sleep is potentially involved. GH secretion has been shown to peak during the onset of night-time sleep, particularly during slow-wave sleep (8-10), and there is evidence of a clinical association between the duration of slow-wave sleep and linear growth. For instance, a meta-analysis revealed that adenotonsillectomy in children with obstructive sleep apnea increased the duration of slow-wave sleep (22) and stimulated linear growth (23) due to increased GH secretion (24, 25). These results may infer a link between increases in slow-wave sleep and enhanced GH secretion, leading to improvement in linear growth in children; however, this scenario has not been experimentally or clinically confirmed. As such, additional studies using healthy pediatric populations are clearly required.

The reasons underlying the lack of association between total sleep duration and subsequent growth may be explained by the insufficient emergence of slow-wave sleep in individuals with increased daytime napping. Although findings from pediatric samples are extremely limited, an observational study involving 46 participants 10 to 23 years of age revealed that multiple naps distributed throughout the day were not as effective in producing slow-wave activity as nighttime sleep (26). In addition, an observational study involving 9 young adults with evening naps reported reduced slow-wave sleep during the following nighttime sleep compared to those without evening naps (27). These results may indicate that reduced slow-wave sleep duration due to insufficient nighttime sleep is not compensated for by an increase in daytime naps.

The strength of this study lies in its use of a large-scale birth cohort with important confounding variables. Herein, we provide evidence suggesting a beneficial role for longer sleep duration at night in linear growth among very young children, which we believe provides a clue to understanding the previously underestimated influence of sleep on linear growth. However, the present study had several limitations. First, owing to the questionnaire-based design of the study, data regarding sleep duration were self-reported. Additionally, data regarding sleep duration are single-day-based; therefore, sleep duration may have been less accurately calculated, creating a bias in the interpretation of the data. Furthermore, sleep quality, including wakefulness, has not been thoroughly investigated. Given the difficulties in defining naps, we instead used nighttime and total sleep duration for the evaluation; therefore, although the current results imply the importance of nighttime sleep, this does not necessarily negate the significance of daytime naps in linear growth, and further assessment is clearly required. Second, information regarding the father's height was not available for nearly one-half of the participants, potentially creating a bias in the interpretation of the data. Finally, a longitudinal evaluation over a longer period was not performed. As sleeping habits change as children grow, a trajectory-based analysis of sleep duration with a longer outcome time will provide more evidence supporting the association between sleep and growth.

In conclusion, this large-scale birth cohort study found a positive association between nighttime sleep duration and subsequent tall stature in young children. Furthermore, total sleep duration was not associated with tall stature, underscoring the importance of nighttime sleep duration for achieving optimal linear growth. Although disrupted sleep patterns, particularly inadequate sleep duration, have long been recognized as detrimental to the health of children, their impact on linear growth has been controversial. Hence, the current study sheds light on the previously underestimated influence of sleep on linear growth and may be useful in informing health-related policies to promote optimal growth and well-being in children.

## Data Availability

Data are unsuitable for public deposition due to ethical restrictions and legal framework of Japan. It is prohibited by the Act on the Protection of Personal Information (Act No. 57 of May 30, 2003, amendment on September 9, 2015) to publicly deposit data containing personal information. Ethical Guidelines for Medical and Health Research Involving Human Subjects enforced by the Japan Ministry of Education, Culture, Sports, Science and Technology and the Ministry of Health, Labour and Welfare also restricts the open sharing of the epidemiologic data. All inquiries about access to data should be sent to: jecs-en@nies.go.jp. The person responsible for handling enquiries sent to this e-mail address is Dr. Shoji F. Nakayama, JECS Programme Office, National Institute for Environmental Studies.

## Abbreviations

GH: growth hormone
HT-SDS: height standard deviation score
JECS: Japan Environment and Children's Study
OR: odds ratio
SDS: standard deviation score
